# A Case of a Giant Liposarcoma at Binh Dan Hospital

**DOI:** 10.7759/cureus.61992

**Published:** 2024-06-09

**Authors:** Huu P Nguyen, Duy Nguyen, An K Vu, Phu V Pham

**Affiliations:** 1 Department of Gastrointestinal Surgery, Binh Dan Hospital, Ho Chi Minh City, VNM; 2 Department of General Surgery, Pham Ngoc Thach University of Medicine, Ho Chi Minh City, VNM

**Keywords:** follow-up, chemotherapy, computed tomography, giant liposarcoma, retroperitoneal sarcomas

## Abstract

Retroperitoneal liposarcomas (RLSs) are uncommon mesenchymal tumors that might present a diagnostic challenge due to their rarity and anatomical location. Despite grossly complete resections, they are commonly linked to a high recurrence rate, necessitating long-term or indefinite follow-up. This report discusses a 59-year-old male patient referred to the Gastrointestinal Department due to chronic abdominal distention, right-sided back pain, and a sizable abdominal mass. The diagnosis was an RLS, and the patient underwent en bloc resection of the mass.

## Introduction

Retroperitoneal sarcomas, which are rare malignant tumors, constitute about 0.15% of all malignancies with an incidence rate of 0.3 to 0.4 per 100,000 individuals in the population [1]. Liposarcomas, accounting for 25%, are the most prevalent type of sarcoma found in the retroperitoneum, succeeded by leiomyosarcomas and undifferentiated pleomorphic sarcomas [2]. These tumors can develop at any age, but they most commonly occur during the sixth and seventh decades of life and show no significant preference for gender or race [3]. The vast potential space in the retroperitoneum allows liposarcomas to reach considerable sizes before compressing nearby structures and causing symptoms [4].

Computed tomography (CT) is the most useful primary method for diagnosing, staging, and conducting preoperative assessments of these tumors [5].

The most reliable predictor of prognosis is achieving a complete surgical resection with clear margins. Numerous studies have identified local tissue invasion as a significant adverse predicting factor for disease survival rate [6]. Pathological analysis, which indicates the differentiation grade, continues to be an essential determinant in the clinical progression and outlook of patients with retroperitoneal liposarcoma [6]. The gold standard for treating primary retroperitoneal liposarcomas (RLSs) is to completely remove the tumor surgically, aiming for negative microscopic margins [6].

The highly aggressive nature of RLSs, coupled with their tendency to recur locally and metastasize distantly despite clear surgical margins, underscores the necessity for adjunctive adjuvant or neoadjuvant therapy [6]. Potential uses for chemotherapy include neoadjuvant cytoreductive therapy, increasing the radiosensitivity of malignancies, and serving as the primary treatment in advanced cases [6]. RLSs often recur within six months to two years following initial surgical resection. Since most recurrences occur locally, it is essential to carefully check the resection site with follow-up imaging for minor alterations. Regional recurrences are sometimes misdiagnosed as fibrosis or surgical scarring. Recurrent liposarcomas typically exhibit imaging characteristics similar to the primary tumor, such as attenuation, and the slightly higher CT attenuation of fat in a recurrent liposarcoma compared to normal retroperitoneal fat assists in diagnosing a recurrence [7].

The National Comprehensive Cancer Network's current surveillance guidelines for retroperitoneal soft-tissue sarcomas advise that patients with low-grade tumors, post-successful resection, should undergo follow-up physical exams and imaging (CT scans of the chest, abdomen, and pelvis) every 3 to 6 months for the first 2 to 3 years, followed by annual check-ups. It is advised that patients with high-grade tumors who had a successful resection undergo follow-up physical examinations and imaging at the same intervals for the first two to three years, then every six months for the next two years, and finally annually after that [8].

## Case presentation

**Figure 1:**
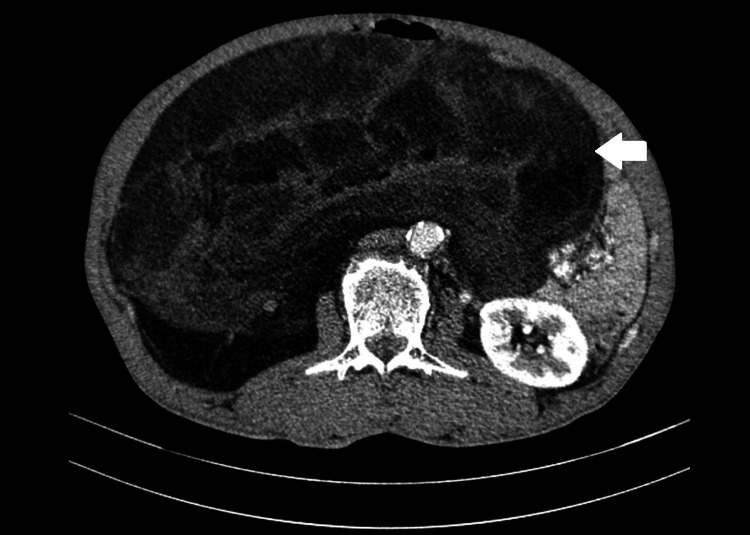
Axial plane of the abdominal CT scan revealed a large right retroperitoneal mass (white arrow)

A 59-year-old man, weighing 70 kg, presented with abdominal pain persisting for three months, accompanied by weight and appetite loss. During the physical examination, his performance was satisfactory; arterial blood pressure registered at 120/70 mmHg, the pulse rate at 80 beats per minute, and oxygen saturation at 98%. Blood tests indicated a white blood cell count of 8x10^3^/mm^3^ and hemoglobin levels of 14 g/dL. The abdominal examination uncovered a large, nontender mass spanning the right and central midline quadrants. An abdominal CT scan revealed a large right retroperitoneal mass comprising various components (fat, solid tissue, and calcium) (Figure 1). No secondary locations were detected.

**Figure 2:**
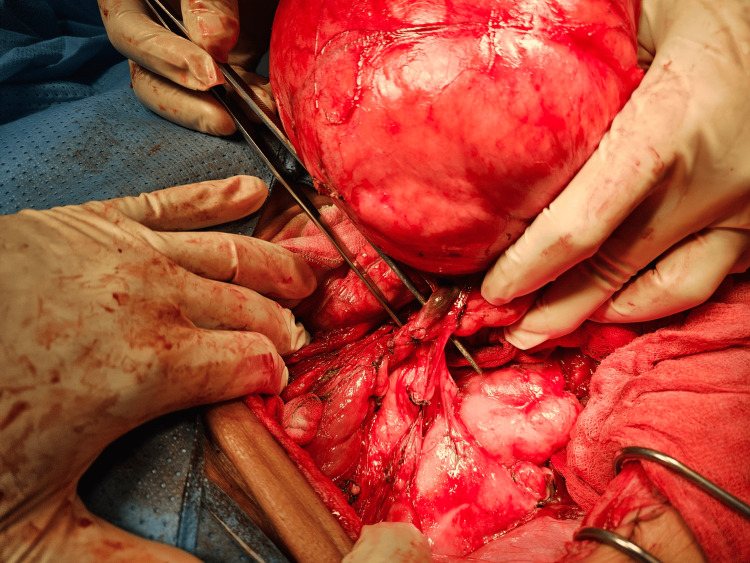
Total excision of the giant retroperitoneal mass without the need to remove other organs

**Figure 3:**
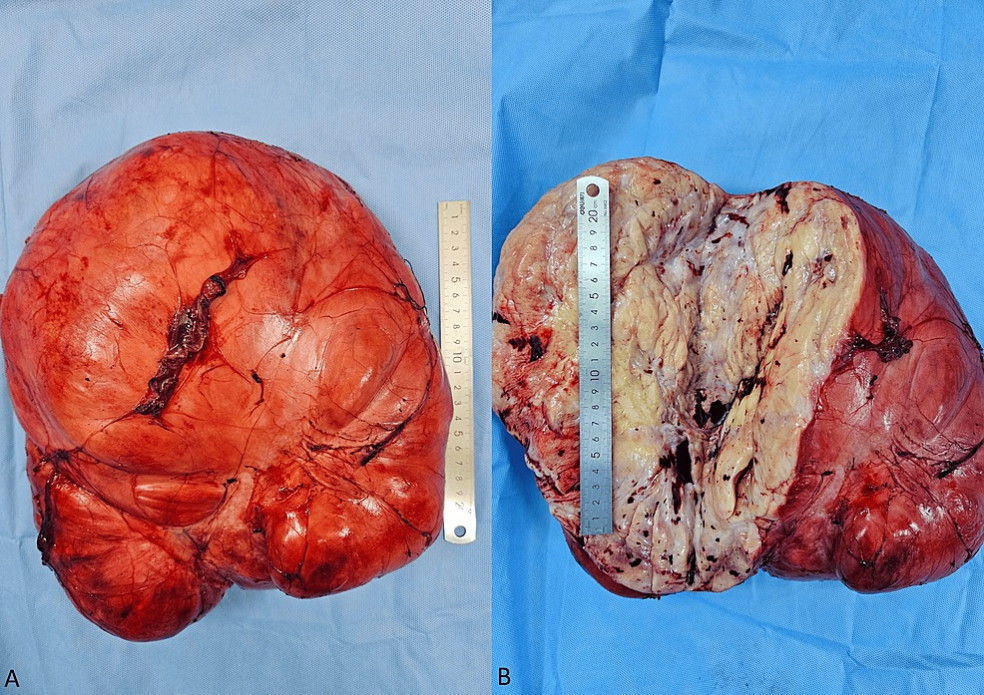
En bloc resected giant liposarcoma (A) and gross image of the tumor (B)

Following a multidisciplinary team meeting, it was decided to proceed with radical surgery without the administration of neoadjuvant chemotherapy. The surgery involved a monobloc resection of the retroperitoneal mass. During the laparotomy, a xipho-pubic incision was made. Upon opening the peritoneum, a sizable retroperitoneal mass approximately 30 cm in size was revealed, stretching from the right hypochondrium to the right iliac fossa. It displaced the right colon and small intestine medially. The mass was exposed following an anteromedial colonic mobilization along Toldt's fascia and complete visualization. An en bloc dissection of the mass was performed with an appendectomy while preserving the right kidney, ureter, and right colon (Figure 2). The surgical specimen's total weight was 10 kg. The tumor measured 34 x 20 x 12 cm (Figure 3).

On postoperative day seven, the patient was clinically well and subsequently discharged. Histopathological analysis confirmed a well-differentiated liposarcoma. The French Federation of Cancer Center Sarcoma Group grading for this tumor was Grade 1 differentiation; Mitotic count: 0; Tumor necrosis: 0.

## Discussion

Liposarcomas weighing over 20kg are exceedingly uncommon and fall into the category of giant liposarcomas [9]. These giant liposarcomas most frequently manifest between the ages of 60 and 70. The incidence rate of RLSs is roughly equal among genders [3]. The definitive diagnostic tools for all cases are CT or magnetic resonance imaging (MRI) [3].

This particular case aligns with other documented instances regarding its nonspecific presentation. Typically, these massive tumors remain symptom-free and grow beyond 15 cm before detection. Symptoms, when present, include discomfort or a detectable lump in the abdomen.

There are four histological variants of RLSs: well-differentiated, myxoid/round cell, pleomorphic, and dedifferentiated. Additionally, they are classified into three grades, I, II, and III, following the grading system established by the French Federation Cancer Centre. The prevalent types are well-differentiated (46%), myxoid/round cell (28%), dedifferentiated (18%), and pleomorphic (8%) [10]. Surgical intervention is paramount for treating well-differentiated RLS since these tumors show negligible response to chemotherapy or radiotherapy [11,12]. According to a study by Zeng et al., the long-term survival rates at 5 and 10 years without complete resection were reported as 16.7% and 8.0%, respectively [12]. Lewis et al. presented data from their institution on 500 cases of RLS, indicating a median survival time of 103 months for patients who underwent complete resection with clean margins versus 18 months for those with incomplete resections [11]. Therefore, striving for complete surgical resection during the initial procedure is crucial.

The decision to opt for primary resection surgery was influenced by CT images showing a giant retroperitoneal encapsulated liposarcoma filling the suitable retroperitoneal space. Adjuvant chemotherapy was ruled out during the multidisciplinary assessment due to the tumor's size [13]. Current clinical management guidelines for sarcomas typically recommend radiotherapy as an adjunct therapy for tumors larger than 5 cm or when surgical resection is incomplete [14]. However, in our case, radiotherapy was not considered due to the complete resection, absence of histological evidence of invasion into other organs, and the extensive size of the tumor site, which would require a broad irradiation field, potentially impairing renal function.

Additionally, the efficacy of perioperative chemotherapy remains controversial; it is suggested for high-risk patients characterized by comorbidities, incomplete resection, organ spread, and cellular dedifferentiation. It has demonstrated more pronounced benefits in patients with limb and chest wall sarcomas than those with retroperitoneal and metastatic disease [15].

Despite the mass's size and irregular edges, complete removal was feasible owing to its full encapsulation, which facilitated separation from adjacent organs. The absence of macroscopic infiltration indicated that a limited resection could be performed, preserving the organs. Histological analysis confirmed a diagnosis of low-grade sarcoma with no evidence of capsular invasion.

In this case, the advanced stage of the disease was solely attributed to the tumor's massive size (>15 cm). Otherwise, the histological type and level of differentiation indicated a low-grade sarcoma with a minimal risk of recurrence. Moreover, the lack of invasion into surrounding organs and complete capsular integrity suggest a favorable prognosis.

## Conclusions

This clinical case demonstrates the challenges of diagnosing and treating a substantial RLS, aiming to minimize risks and enhance rehabilitation. The tumor size does not preclude the possibility of complete resection, even in the case of giant RLSs. Regardless of their size, such tumors can be excised in a multidisciplinary setting by an experienced team specializing in sarcoma treatment at a tertiary center. As demonstrated in our case, multidisciplinary collaboration is essential for treating this type of tumor, involving surgeons, pathologists, imaging experts, radiation therapists, and medical oncologists.
